# Prevalence, Risk Factors and Intervention for Depression and Anxiety in Pulmonary Hypertension: A Systematic Review and Meta-analysis

**DOI:** 10.3389/fmed.2022.765461

**Published:** 2022-02-17

**Authors:** Aaron Shengting Mai, Oliver Zi Hern Lim, Yeung Jek Ho, Gwyneth Kong, Grace En Hui Lim, Cheng Han Ng, Cyrus Ho, Roger Ho, Yinghao Lim, Ivandito Kuntjoro, Edgar Tay, James Yip, Nicholas W. S. Chew, Ting-Ting Low

**Affiliations:** ^1^Yong Loo Lin School of Medicine, National University of Singapore, Singapore, Singapore; ^2^Lee Kong Chian School of Medicine, Nanyang Technological University, Singapore, Singapore; ^3^Department of Psychological Medicine, Yong Loo Lin School of Medicine, National University of Singapore, Singapore, Singapore; ^4^Deparment of Cardiology, National University Heart Centre, Singapore, Singapore

**Keywords:** depression, anxiety, risk factors, prevalence, pulmonary hypertension, interventions

## Abstract

**Background:**

Current guidelines recommend psychological support for patients with pulmonary hypertension suffering from psychological adversity. However, little is known about the prevalence and risk factors of depression and anxiety in patients with pulmonary hypertension (PH).

**Methods:**

Medline and Embase were searched from inception to 6 May 2021. Meta-analysis of proportions using the generalized linear mixed model was conducted to analyze the pooled prevalence rates of depression and anxiety in PH patients. Risk factors for depression and anxiety in PH patients were evaluated using meta regression.

**Results:**

A total of 24 studies involving 2,161 PH patients were included. The pooled prevalence of depression in PH was 28.0% (95% CI: 20.5–36.8) and pooled prevalence of anxiety was 37.1% (95% CI: 28.7–46.4). There was a significantly higher prevalence of anxiety (*p* = 0.0013) amongst PH patients in Asia (61.1%) compared to Europe (40.3%) and North America (22.9%). In terms of risk factors, congenital heart disease-related pulmonary arterial hypertension (PAH-CHD) were significantly associated with both depression (OR: 1.68, 95% CI: 1.27–2.23, *p* = 0.024) and anxiety (OR: 1.63, 95% CI: 1.45–1.83, *p* = 0.002). On the other hand, chronic thromboembolic pulmonary hypertension (CTEPH, OR: 1.18, 95% CI: 1.10–1.26, *p* = 0.004) was significantly associated with depression, whereas worse pulmonary vascular resistance (β: 0.30, 95% CI: 0.09–0.52, *p* = 0.005) and cardiac index (β: −0.96, 95% CI: −1.58 to −0.35, *p* = 0.002) were significantly correlated with anxiety.

**Conclusion:**

The prevalence of anxiety and depression in PH patients is alarmingly high, with an increased prevalence of anxiety in Asia compared to Europe or North America. Psychological support is warranted for patients with PH, particularly those with underlying congenital heart disease, CTEPH, and severe disease.

**Systematic Review Registration:**

CRD42021251733.

## Introduction

Pulmonary hypertension (PH) is a hemodynamic condition with resting mean pulmonary artery pressure ≥20 mm Hg assessed by right heart catheterization. This revised definition has been recently introduced at the 6^th^ World Symposium on PH, with a reduced mean pulmonary arterial pressure (mPAP) criterion from ≥25 mm Hg to >20 mm Hg (1). However, most PH prevalence studies have adopted the conventional threshold of mPAP ≥25 mm Hg and this may potentially result in an underestimated prevalence. PH is classified into five groups based on its clinical presentation, pathological features and hemodynamic findings, that will influence the treatment strategy. Approved therapies are available only for pulmonary arterial hypertension (PAH) and chronic thromboembolic PH (CTEPH), where treatment in PH-centers is recommended (2). Currently, there are no approved therapies for patients with PH associated with left heart disease (PH-LHD), PH associated with lung disease (PH-lung), and miscellaneous causes (PH-misc). Selected patients may benefit from trials of individualized treatment decisions in expert centers (2).

PAH is a serious condition that is characterized by progressive obliteration of pulmonary vessels and is frequently fatal. Even with targeted therapies, the median survival rates are 86 and 61% at one and five years after diagnosis respectively (3). Patients suffer from dyspnea, fatigue, syncope, and eventually right heart failure. The rarity of the disease, guarded prognosis, coupled with high treatment costs have impacted patient's lives beyond physical limitations. Depression and anxiety are increasingly being recognized as profoundly affecting the health-related quality of life of these patients (4).

Few studies examine the impact of depression and anxiety in patients with PH, with prevalence rates reported widely from 8–55% and 19–48% respectively in PAH (5–9). The association between characteristics, risk factors, and etiologies of PH with depression and anxiety remains unclear. This systematic review and meta-analysis will be the first, to the best of our knowledge, to examine the prevalence, risk factors, and interventions for depression and anxiety in patients with PH, especially PAH and CTEPH.

## Methods

### Search Strategy

This review was conducted with reference to the Preferred Reporting Items for Systematic Reviews and Meta-Analyses (PRISMA) guidelines (10). Medline and Embase databases were accessed, and relevant papers were identified from inception up to 6 May 2021. Keywords and MeSH terms synonymous with “Pulmonary Hypertension”, “Depression” and “Anxiety” were used in the identification of suitable articles in the initial search. The full search used for Medline was: ((pulmonary^*^ adj2 hypertensi^*^)tw. or exp Hypertension, Pulmonary/) AND ((exp Behavioral Symptoms/or exp Psychological Distress/or exp “Quality of Life”/) or exp Depression/or exp anxiety/or (depress^*^ or anxiety^*^ or anxiou^*^).tw. References were imported into Endnote X9 for the initial sieve and duplicates were removed. Additionally, references of previous related reviews were screened to ensure a comprehensive search (11, 12).

### Study Selection and Extraction

Eligibility for inclusion was determined by two authors (ASM and OZHL) who screened articles from the initial sieve, with a third independent author involved in the resolution of disputes. Retrospective and prospective cohort studies, case-control, randomized controlled trials, interventional studies, and cross-sectional studies were considered for inclusion, while case reports, case series, editorials, systematic reviews, meta-analyses, and commentaries were excluded. Only English-language articles were considered. Inclusion criteria consisted of: (i) studies assessing the prevalence, risk factors, and interventions of depression and anxiety (ii) patients with a diagnosis of PH made via right heart catheterization, imaging techniques (pulmonary angiography, echocardiogram). (iii) Diagnosis of depression was identified through patient responses from self-rated scales such as Hospital Anxiety and Depression Scale (HADS), Patient Health Questionnaire (PHQ), Depression Anxiety Stress Scales (DASS-21), Quick Inventory of Depressive Symptomatology (QIDS), and Beck's Depression Inventory scale (BDI) or patients' medical history. Similarly, self-rated scales of anxiety comprising Hospital Anxiety and Depression Scale (HADS), Depression Anxiety Stress Scales (DASS-21), Patient Health Questionnaire (PHQ), General Anxiety Disorder-7 (GAD-7) and Beck Anxiety Inventory scale (BAI) and patients' medical history were utilized in the diagnosis of anxiety. Pediatric studies were excluded from the analysis.

Relevant data were extracted from included articles by two blinded authors (AMS, OLZH). The domains extracted included baseline demographics (author, year, country of study, sample size, mean age, gender, ethnicity, and employment status), baseline clinical data (PH classification, hemodynamics, functional status, oxygen use), the prevalence of depression and anxiety, as well as interventions and associated outcomes. The main outcomes of interest were the prevalence, risk factors of depression and anxiety in PH patients, and its interventions. Estimated values of the mean and standard deviation were derived using formulas devised by Wan et al. and Furukawa et al. when they were not provided (13, 14).

### Statistical Analysis and Quality Assessment

The statistical analysis of included articles was performed with a single-arm proportional meta-analysis and meta-regression in RStudio (version 1.3.1093). Statistical significance was established for outcomes with a *p-*value < 0.05. A single-arm proportional meta-analysis was performed using the generalized linear mixed model (GLMM) with Clopper-Pearson intervals to stabilize the variance (15). A random-effects model was used to generalize findings beyond the included studies as a more robust estimate of the true prevalence regardless of heterogenicity scores (16).

Additional subgroup analysis was considered based on the continent and income level of countries. Income level was defined using the World Bank's classification of Countries by Income (17). Subsequently, a sensitivity analysis was conducted to summarize the rates of depression and anxiety in patients with PAH or CTEPH. As with previous meta-analyses on depression and anxiety (15, 18), a separate subgroup analysis based on the diagnostic tool was performed to reduce heterogeneity. Conventional measures of statistical heterogeneity can be inaccurate in prevalence-based meta-analysis (19) resulting in an I^2>^ 90% in prevalence-based meta-analysis (20). Regardless, quantification of heterogeneity was done via I^2^ and Cochran Q test values, where an I^2^ >40% or a Cochran Q test with a *p*-value of >0.10 was considered significant for heterogeneity (21).

To explore risk factors that could relate to a diagnosis of depression or anxiety in PH patients, a mixed-effects meta-regression was used to quantify continuous variables that had a positive or negative coefficient relationship with depression or anxiety. For binary variables, a generalized linear model was conducted in the binomial family and logit link with inverse variance weightage, after which the coefficient was then exponentiated to obtain the odds ratio (OR) (22). Binary variables were considered using a 10-fold increase in odds. Despite the use of antidepressants and anxiolytics being a potential protective factor against depression and anxiety, there were not enough studies reporting this data for meta-regression to be conducted. Lastly, systematic reporting was used to summarize the evidence of literature surrounding non-pharmacological interventions to decrease depression or anxiety in PH patients.

As the analysis was primarily conducted with proportion-based meta-analysis, publication bias was not assessed with the lack of a suitable tool in single-arm meta-analysis to assess publication bias and a small quantity of included studies (23). Quality assessment of included articles was done with the Joanna Briggs Institute (JBI) Critical Appraisal Tool (24). The JBI is the most widely used tool in prevalence meta-analysis to assess the quality of included articles based on the rigor of methodology and appropriateness of statistical analysis (19).

## Results

### Summary of Included Articles

**Figure 1:**
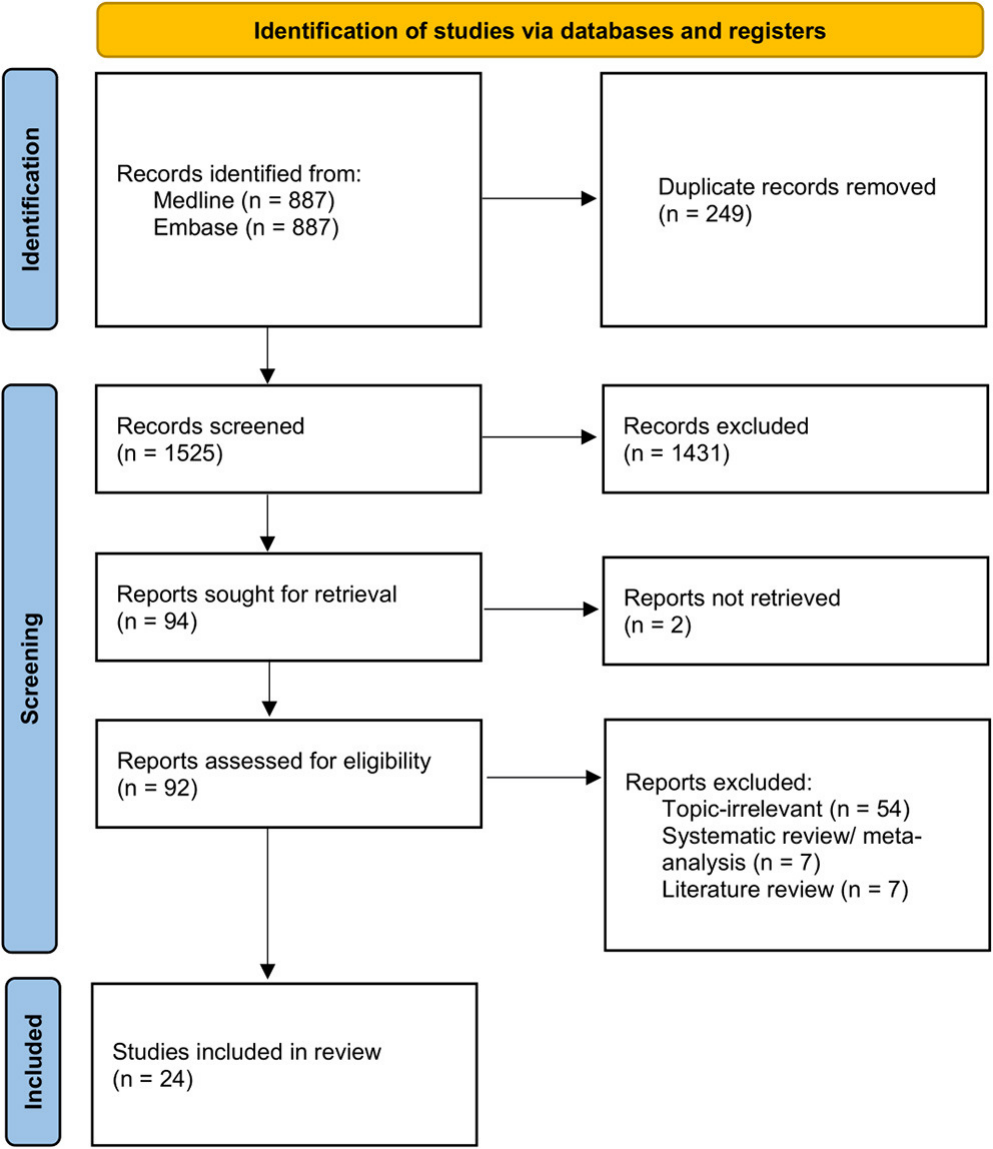
PRISMA flowchart of included studies.

A total of 1,774 references were identified in the initial search strategy, with 1,525 references screened after the removal of duplicates. A full-text review was carried out for 43 papers with 24 articles involving 2,161 patients with PH included in the final analysis (Figure 1). Depression was identified in a total of 21 studies using various scales such as HADS (4, 6, 25–30), DASS-21 (9, 31), PHQ (5, 8, 32–34), QIDS (35), and BDI scale (7, 36, 37). In two studies, depression was identified through patients' disclosure of medical history (38, 39). As for anxiety, a total of 16 studies were evaluated by utilizing scales such as HADS (4, 6, 25–30), DASS-21 (9, 31), PHQ (5, 33), GAD-7 (32, 34), and BAI (7). Anxiety was identified through patients' medical history in 1 study (39).

Twelve studies had patients with PAH (6, 7, 9, 25, 28–32, 34, 36, 38). PH-LHD, PH-lung, CTEPH and PH-misc were present in four (9, 31, 39, 40), five (25, 31, 36, 39, 40), ten (4, 9, 25, 27, 31, 32, 36, 37, 39, 40) and three studies (31, 39, 40) respectively. A diagnosis of PH was conducted through right heart catheterization (RHC) in thirteen studies (6–9, 25, 27, 28, 30–32, 36, 37, 39), imaging techniques only in one study (35), and both RHC and imaging techniques in seven studies (4, 5, 26, 29, 33, 34, 38).

Two studies evaluated interventions for both depression and anxiety (30, 41), while another two only evaluated interventions for either depression or anxiety (42, 43). Subgroup analysis by income level was conducted for the prevalence of depression and anxiety for 18 high-income countries (4–9, 26–29, 31, 33–39) and 3 middle-income countries (25, 30, 32). The income levels of the countries were determined using the World Bank's classification of Countries by Income (17). A summary of included articles can be found in Table 1.

**Table 1:** Summary of included studies.

**References**	**Country**	**Study design**	**Sample size/N (pulmonary hypertension)**	**Mean age/years**	**Pulmonary hypertension subgroup/N**	**Depression/anxiety events/N**	**Tool (depression/ anxiety)**	**Definition (depression/anxiety)**	**Quality**
Nakazato et al. (25)	Brazil	Cross-sectional	20	44.3 ± 13.2	PAH 20	3/6	HADS-D/ HADS-A	HADS>=8/ HADS>=8	8/8
Von Visger et al. (43)	USA	Randomized controlled trial	14	58.26 ± 11.07	PAH 8, PH-LHD 5, PH-lung 1	-/-	-/VAS-A	-	8/11
Zhou et al. (32)	China	Cohort	98	48.48 ± 14.3	PAH 36, CTEPH 62	56/51	PHQ/ GAD-7	PHQ>=5/GAD>=5	10/11
Tajima et al. (38)	Japan	Retrospective	229	58.69 ± 11.98	CTEPH 229	10/-	Medical history	History of psychiatric attendances and ongoing use of psychiatric medication/-	9/11
Lo et al. (39)	Canada	Cross-sectional	30	51.7 ± 17.83	PAH 30	3/4	Medical history	-/-	8/8
Aguirre-Camacho et al. (26)	Spain	Cross-sectional	64	49.8 ± 13.73	PAH 57, CTEPH 7	14/23	HADS-D/ HADS-A	HADS>=8/HADS>=8	6/8
Halimi et al. (27)	France	Cohort	55	57.8 ± 15.3	PAH 43, CTEPH 12	23/32	HADS-D/ HADS-A	HADS>=8/HADS>=8	10/11
Vanini et al. (41)	Italy	Randomized controlled trial	70	48 ± 10	CTEPH 70	-/-	HADS-D/HADS-A	HADS >7/HADS >7	11/11
Von Visger et al. (33)	USA	Cross-sectional	108	56.33 ± 14.28	PAH 108	27/12	PHQ/PHQ	PHQ>=5/PHQ>=5	6/8
Funabashi et al. (35)	Japan	Cross-sectional	40	58.13 ± 17.29	CTEPH 40	15/-	QIDS/-	QIDS>=6/-	6/8
Pfeuffer et al. (4)	Germany	Cross-sectional	93	69.5 ± 11.25	PAH 70, CTEPH 23	34/33	HADS-D/ HADS-A	HADS>=8/HADS>=8	6/8
Amedro et al. (28)	France	Cross-sectional	208	42.6 ± 15.8	PAH 208	19/64	HADS-D/ HADS-A	HADS>=8/HADS>=8	8/8
Matura et al. (42)	USA	Randomized controlled trial	10	50.1 ± 13.5	PAH 10	-/-	PHQ/-	PHQ >=4	8/11
Somaini et al. (29)	Switzerland	Cohort	131	64.33 ± 17.99	PAH 91, PH-lung 10, CTEPH 30	43/45	HADS-D/ HADS-A	HADS>5/HADS>5	9/11
Tartavoulle et al. (31)	USA	Cross-sectional	166	52 ± 13.7	PAH 154, CTEPH 1, PH-LHD 6, PH-lung 3, PH-misc 2	54/103	DASS-21/ DASS-21	DASS>=10/DASS>=8	6/8
Li et al. (30)	China	Randomized controlled trial	114	54.03 ± 12.54	PAH 114	68/79	HADS-D/ HADS-A	HADS>8/ HADS>8	9/13
Vanhoof et al. (9)	Belgium	Cross-sectional	101	55.4 ± 16.4	PAH 101	32/48	DASS-21/DASS-21	DASS>=10/ DASS>=8	6/8
Harzheim et al. (34)	Germany	Prospective cohort	158	56 ± 16	PAH 129, CTEPH 20, PH-LHD 1, PH-lung 6, PH -misc 2	91/72	PHQ/ GAD-7	PHQ>=5/ GAD>=5	10/11
Batal et al. (36)	USA	Cross-sectional	40	52.5 ± 11.7	PAH 31, CTEPH 4, PH-lung 5	17/-	BDI/-	BDI>=10	6/8
McCollister et al. (8)	USA	Cohort	100	–	PAH 100	55/-	PHQ/-	PHQ>=5	6/11
Looper et al. (37)	Canada	Cross-sectional	52	61.2 ± 14	PAH 28, CTEPH 3, PH-LHD 21	11/-	BDI/-	BDI>=17	8/8
White et al. (7)	USA	Cross-sectional	46	48.2 ± 11.8	PAH 46	12/9	BDI/ BAI	BDI>=10/ BAI>=8	8/8
Shafazand et al. (6)	USA	Cross-sectional	53	47 ± 11	PAH 53	4/11	HADS-D/ HADS-A	HADS>=8/ HADS>=8	6/8
Löwe et al. (5)	Germany and Austria	Cross-sectional	164	47.8 ± 12.7	PAH 128, CTEPH 16, PH-lung 6, PH-LHD 1, PH-misc 13	37/66	PHQ/ PHQ	PHQ>=5/ PHQ>=5	8/8

*PAH, pulmonary arterial hypertension; PH-LHD, pulmonary hypertension related to left heart disease; PH-lung, pulmonary hypertension related to lung disease; CTEPH, chronic thromboembolic pulmonary hypertension; PH-misc, pulmonary hypertension related to miscellaneous causes; HADS, Hospital Anxiety Depression Scale; VAS-A, Visual Analog Scale-Anxiety; PHQ, Patient Health Questionnaire; QIDS, Quick Inventory of Depressive Symptomatology; GAD-7, Generalized Anxiety Disorder-7; DASS-21, Depression Anxiety Stress Scale-21; BDI, Beck Depression Inventory; BAI, Beck Anxiety Inventory; -, no data reported*.

### Depression

#### Prevalence of Depression PH Patients

**Figure 2:**
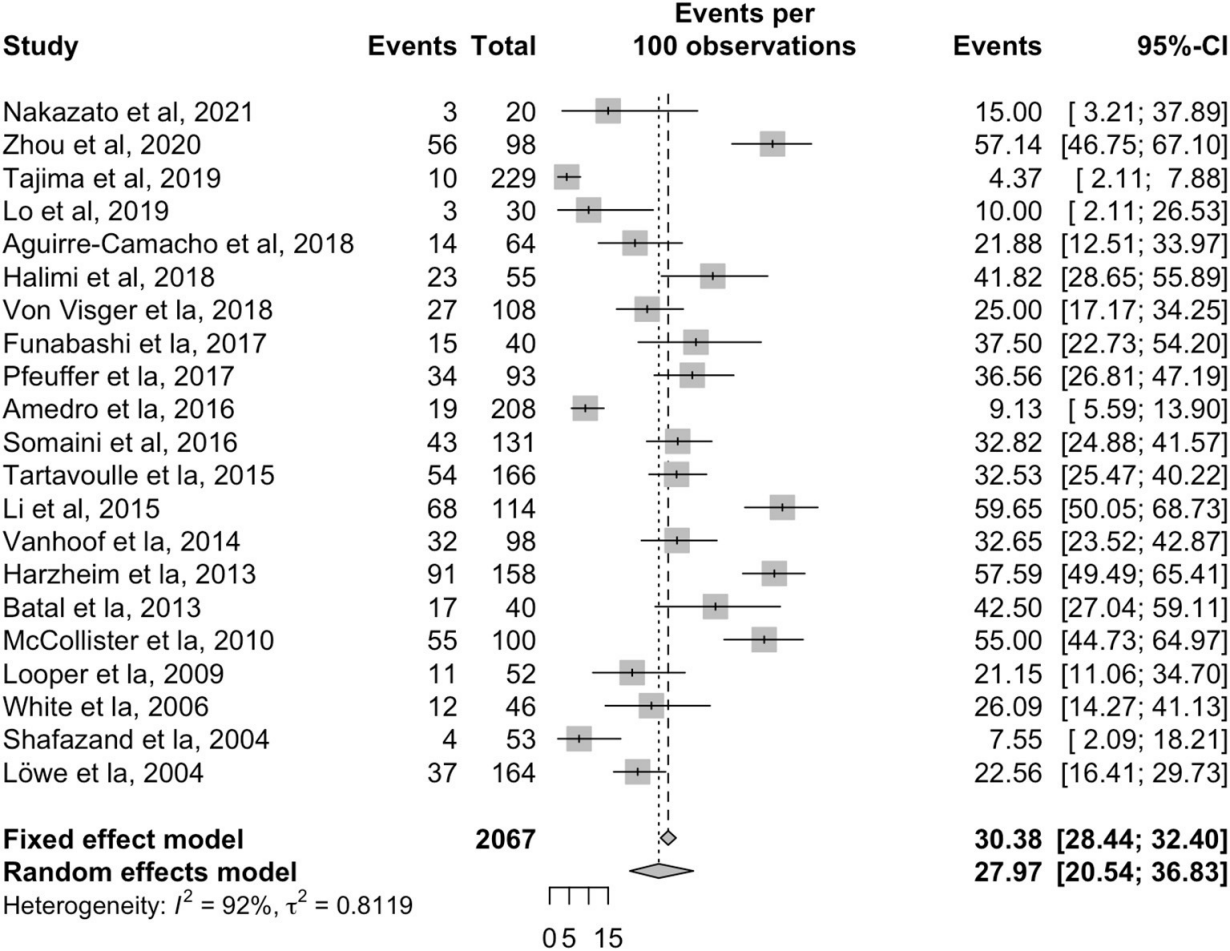
Forest plot for the pooled prevalence of depression in patients with pulmonary hypertension.

The overall pooled prevalence for depression in patients with PH was 28.0% (95% CI: 20.5–36.8, Table 2, Figure 2) in 2,067 individuals. Sensitivity analyses were conducted for both PAH and CTEPH populations for depression. The pooled prevalence of depression in 905 PAH patients was 25.6% (95% CI: 16.3–37.7) across eleven articles (4, 6–9, 25, 27, 28, 30, 33, 39) while 292 CTEPH patients had a pooled prevalence of depression of 24.1% (95% CI: 5.7–62.3) across three articles (4, 35, 38). A subgroup analysis was conducted to explore regional and income differences, but no significant differences were found between the middle and high-income countries (*p* = 0.127) or between geographical regions (*p* = 0.83). A final subgroup analysis was conducted to account for the variations in the diagnostic tools. The prevalence of depression was 25.1% with HADS-D (95% CI: 14.7–39.5); 29.1% with BDI (95% CI: 20.1–40.0); 32.6% with DASS-21 (95% CI: 27.2–38.5); 37.5% with QIDS (95% CI: 24.0–53.2); 23.3% with PHQ (95% CI: 8.7–49.1).

**Table 2:** Prevalence and subgroup analyses of depression and anxiety in patients with PH.

	**Depression**					**Anxiety**				
	**No. of articles**	**Total sample size**	**Effect size**	**95%CI**	***P*** **value**	**No. of articles**	**Total sample size**	**Effect size**	**95%CI**	***P*** **value**
**Prevalence**										
Overall	21	2,067	28.0	20.5–36.8	–	16	1,600	37.1	28.7–46.4	–
PAH only	11	905	25.6	16.3–37.7	–	10	805	31.5	20.6–44.7	–
CTEPH only	3	292	24.1	5.8–62.3	–	–	–	–	–	–
**Region**										
Asia	4	481	32.6	10.8–66.0	0.830	2	212	61.1	48.6–62.3	0.0013
North America	8	595	25.7	16.5–37.9	0.830	5	399	22.9	11.1–41.4	0.0013
Europe	8	971	29.889	20.4–41.4	0.830	8	969	40.3	35.2–45.7	0.0013
**Income level**										
Middle income	3	232	44.8	22.9–68.9	0.127	3	232	53.2	35.7–70.0	0.059
High income	18	1,835	25.8	18.7–34.5	0.127	13	1,368	34.1	25.8–43.5	0.059
**Diagnostic tool**										
HADS-D/A	8	738	25.2	14.7–39.5	<0.001	8	734	39.2	29.0–50.5	<0.001
PHQ	5	628	42.4	28.3–57.8	<0.001	2	268	23.3	8.7–49.1	<0.001
QIDS/GAD-7	1	40	37.5	24.0–53.2	<0.001	2	256	48.0	42.0–54.2	<0.001
DASS-21	2	264	32.6	27.2–38.5	<0.001	2	266	55.8	45.9–65.2	<0.001
BDI/BAI	3	138	29.0	20.1–40.0	<0.001	1	46	19.6	10.5–33.5	<0.001

*PH, pulmonary hypertension; PAH, pulmonary arterial hypertension; CTEPH, chronic thromboembolic pulmonary hypertension; HADS, Hospital Anxiety Depression Scale; PHQ, Patient Health Questionnaire; QIDS, Quick Inventory of Depressive Symptomatology; GAD-7, Generalized Anxiety Disorder-7; DASS-21, Depression Anxiety Stress Scale-21; BDI, Beck Depression Inventory; BAI, Beck Anxiety Inventory; -, no data reported*.

#### Risk Factors for Depression

Meta-regression was performed to identify risk factors related to a depression diagnosis (Table 3). PAH related to congenital heart disease (PAH-CHD) (OR: 1.68, 95% CI: 1.27–2.23, *p* = 0.024) and CTEPH (OR: 1.18, 95% CI: 1.10–1.26, *p* = 0.004) were significantly associated with increased odds for depression. There were no significant associations between age, ethnicity, gender, or functional status with depression in patients with PH.

**Table 3:** Risk factors of depression and anxiety in patients with PH.

	**Depression**			**Anxiety**		
	**Effect size**	**95% CI**	***p*-value**	**Effect size**	**95% CI**	***p*-value**
**Demographics**						
Age	β 0.038	−0.023–0.098	0.220	β 0.014	−0.041–0.069	0.610
White	OR 1.352	0.847–2.157	0.348	OR1.221	0.942–1.584	0.295
Female	OR 0.938	0.681–1.293	0.746	OR1.071	0.715–1.606	0.783
Married	OR 0.880	0.773–1.003	0.169	OR 1.203	0.771–1.877	0.532
Unemployed	OR 0.961	0.900–1.027	0.396	-	-	-
**Diagnosis**						
PAH Total	OR 0.900	0.795–1.018	0.179	OR 0.936	0.848–1.034	0.292
PAH – Idiopathic	OR 0.964	0.779–1.192	0.782	OR 0.887	0.793–0.991	0.135
PAH – CTD	OR 1.132	0.692–1.850	0.689	OR 1.141	0.669–1.945	0.699
PAH - Portal HTN	OR 1.330	0.986–1.794	0.215	-	-	-
PAH – CHD	OR 1.678	1.265–2.225	0.024	OR 1.631	1.453–1.831	0.002
CTEPH	OR 1.175	1.098–1.257	0.004	OR 0.980	0.846–1.136	0.830
PH-Lung	OR 1.115	0.572–2.170	0.806	-	-	-
**Severity**						
WHO I-II	OR 0.879	0.745–1.038	0.271	OR 0.981	0.853–1.128	0.838
WHO III-IV	OR 1.124	0.980–1.290	0.235	OR 1.019	0.905–1.148	0.807
Oxygen Use	OR 1.162	0.872–1.550	0.418	OR 0.914	0.724–1.154	0.547
6MWD (m)	β −0.003	−0.022–0.016	0.764	β −0.009	−0.019–0.001	0.086
mPAP (mmHg)	β −0.022	−0.092–0.049	0.548	β −0.011	−0.062–0.040	0.681
PVR (Wood units)	β 0.081	−0.204–0.367	0.577	β 0.303	0.091–0.515	0.005
RAP (mmHg)	β −0.049	−0.174–0.076	0.439	β −0.091	−0.295–0.112	0.379
CI (L/min/m^2^)	β −1.809	−4.087–0.469	0.120	β −0.963	−1.582–−0.345	0.002

*PH, pulmonary hypertension; PAH, pulmonary arterial hypertension; CTD, connective tissue disease; Portal HTN, portal hypertension; CHD, congenital heart disease; CTEPH, chronic thromboembolic pulmonary hypertension; PH-lung, pulmonary hypertension related to lung disease; WHO, World Health Organization; 6MWD, 6-minute walk distance; mPAP, mean pulmonary artery pressure; PVR, pulmonary vascular resistance; RAP, right atrial pressure; CI, cardiac index; - no data reported*.

### Anxiety

#### Prevalence of Anxiety

**Figure 3:**
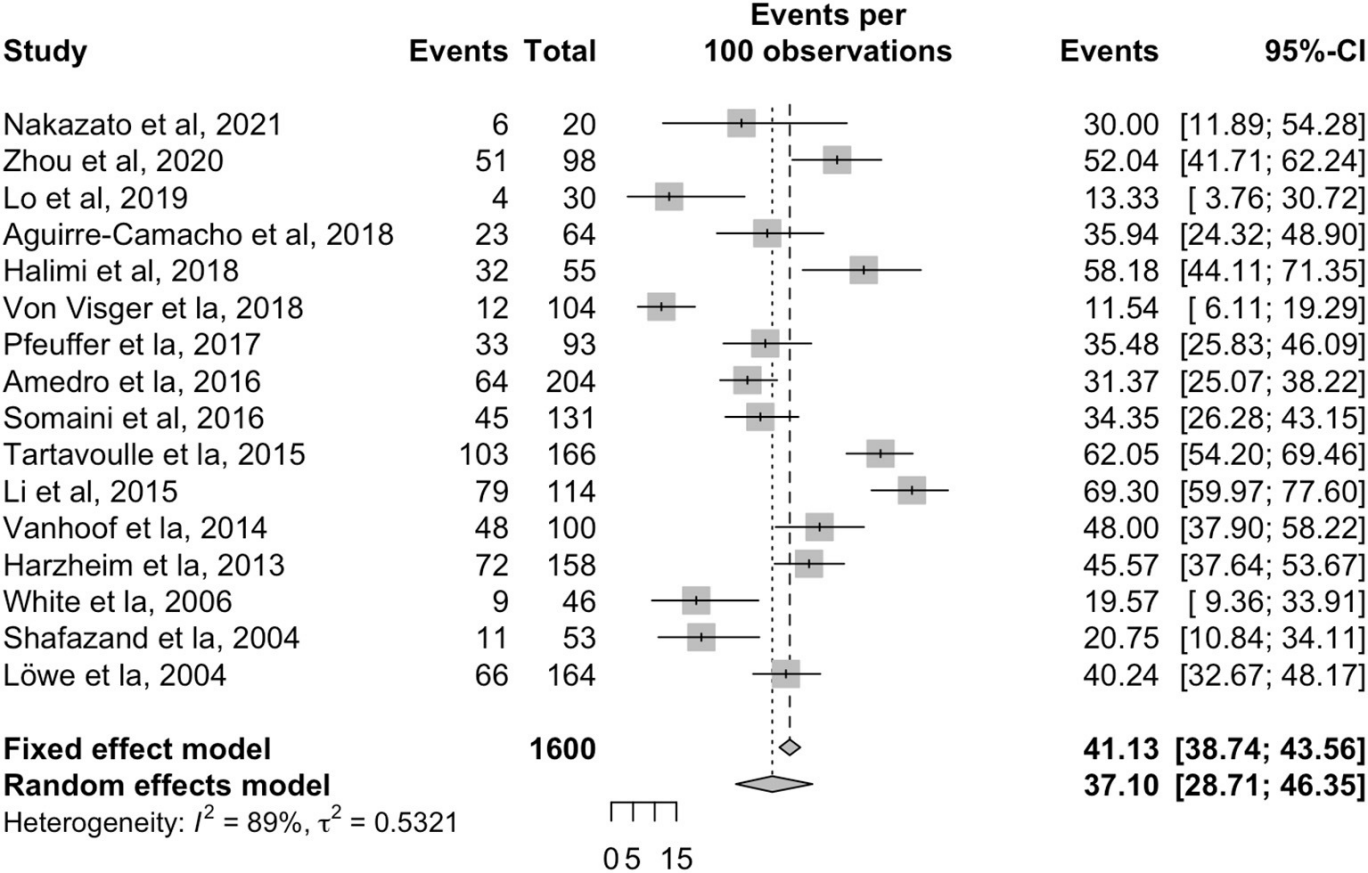
Forest plot for the pooled prevalence of anxiety in patients with pulmonary hypertension.

**Figure 4:**
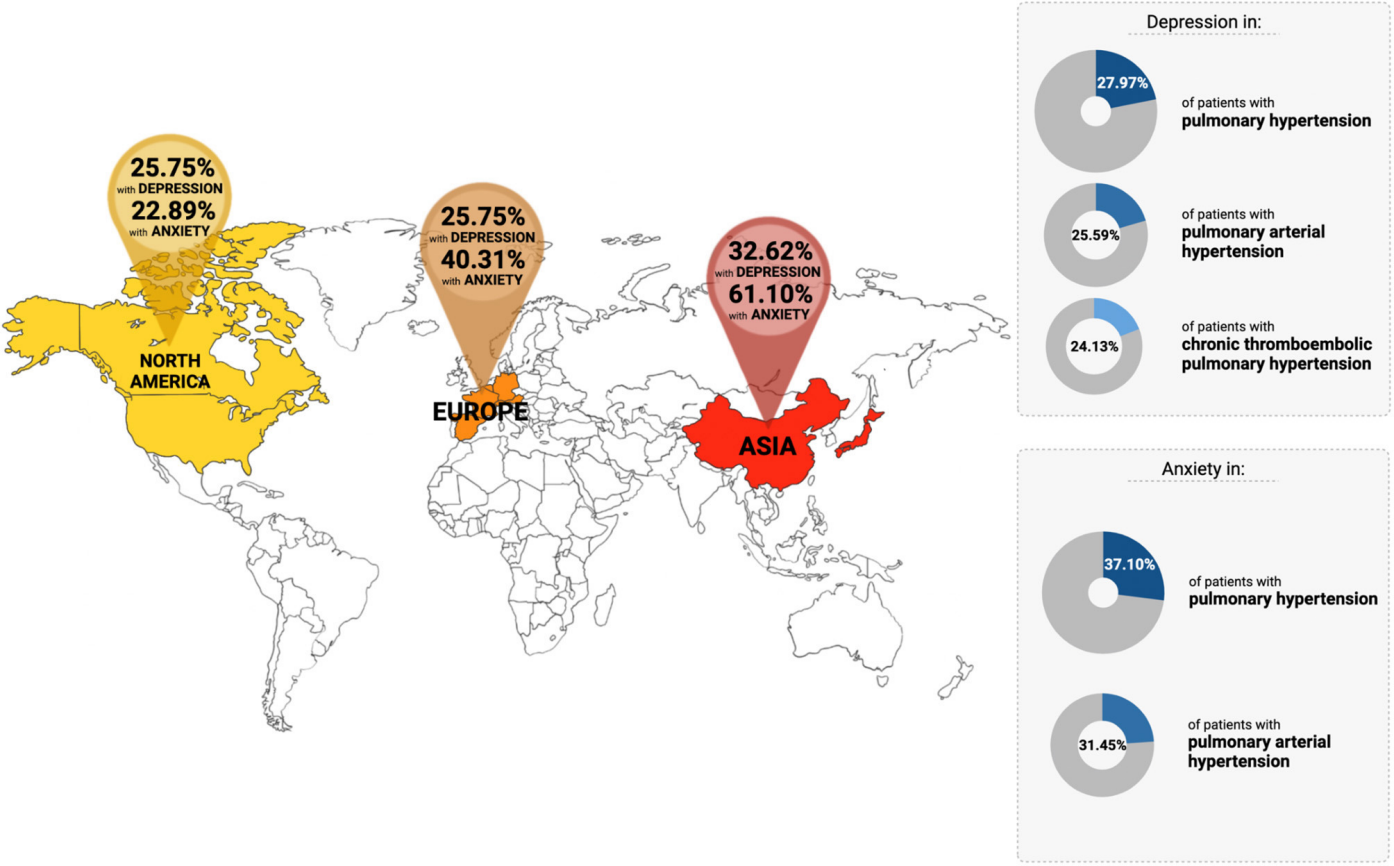
Overview of the prevalence of depression and anxiety in patients with pulmonary hypertension across the countries.

Overall pooled prevalence for anxiety in patients with PH was 37.1% (95% CI: 28.7–46.4, Table 2, Figure 3) in 1,600 individuals. A sensitivity analysis was conducted, with the pooled prevalence of anxiety in 805 PAH patients found to be 31.5% (95% CI: 20.6–44.7) across ten articles (4, 6, 7, 9, 25, 27, 28, 30, 33, 39). There was inadequate data on anxiety in the CTEPH population for a sensitivity analysis to be conducted. Subgroup analyses were conducted to account for regional and income differences. Significant differences were found between geographical regions (*p* = 0.0013). The pooled prevalence of anxiety in studies conducted within Asia was 61.1% (95% CI: 48.6–62.4) compared to 40.3% (95% CI: 35.2–45.7) in Europe, and 22.9% (95% CI: 11.1–41.4) in North America. No significant differences were found between the middle and high-income countries (*p* = 0.059). A final subgroup analysis was conducted to account for differences between diagnostic tools. Prevalence of anxiety was 19.6% with BAI (95% CI: 10.5–33.5); 23.3% with PHQ (95% CI: 8.7–49.1); 39.2% with HADS-A (95% CI: 29.0–50.5); 48.1% with GAD-7 (95% CI: 42.0–54.2) and 55.8% with DASS-21 (95% CI: 45.9–65.2). A summary of the prevalence of depression and anxiety can be found in Figure 4.

#### Risk Factors for Anxiety

Table 3 summarizes the risk factors related to anxiety in PH patients. PAH-CHD was identified as a significant risk factor (OR: 1.63, 95% CI: 1.45–1.83, *p* = 0.002). Pulmonary vascular resistance (β: 0.30, 95% CI: 0.09 to 0.52, *p* = 0.005) and cardiac index (β: −0.96, 95% CI: −1.58 to −0.35, *p* = 0.002) were found to significantly correlate with anxiety. No associations between anxiety and age, ethnicity, gender, or functional status were found.

### Interventions

A total of three studies examined interventions for depression and anxiety in PAH patients (Table 4). Li et al. reported a significant improvement in both depressive and anxiety symptoms after progressive muscle relaxation (PMR) techniques (*p* < 0.05) (30). A 2017 paper by Matura et al. revealed that slow-paced respiration therapy reduced the severity of depression, with PHQ-8 scores decreasing from a baseline mean of 4.2 to a follow-up mean of 2.9 after eight weeks of therapy (42). Reduction in anxiety symptom severity before and after weekly urban Zen integrative therapy (UZIT) was reported by Von Visger et al. (*p* < 0.0001) (43). Another study evaluated an intervention for both depression and anxiety in CTEPH. Vanini et al. reported that using moderate hypothermic circulatory arrest (MHCA) during pulmonary endarterectomy (PEA) significantly reduced depressive and anxiety symptoms in patients, as measured by a significant decrease in HADS-D score (*p* = 0.002) and HADS-A score (*p* < 0.001) respectively (41). None of the included studies described pharmacological interventions for anxiety and depression.

**Table 4:** Summary of interventional studies.

**Population**	**References**	**N for intervention/control**	**Intervention**	**Depression/anxiety**	**Outcomes**
PAH	Li et al. (30)	55/59	Progressive muscle relaxation	Intervention for depression and anxiety	PMR group showed significant improvement in anxiety, depression, overall QOL and QOL-MCS, but not QOL-PCS or 6MWD (*p* < 0.05). Control group showed no significant improvement in any of the variables. The control group showed no significant changes in distribution of the HADS-Anxiety and the HADS-Depression scores after intervention compared with that at baseline. In contrast, the number/proportion of patients with an anxiety (*p* < 0.01) or depression score (*p =* 0.01) less than 8 markedly increased in the PMR group after intervention compared with that at baseline.
	Matura et al. (42)	10/0	Slow-paced respiration therapy (using the RESPeRATE device)	Intervention for depression only	Slow-paced respiration therapy was found to decrease the severity of depression in the population. PHQ-8 scores decreased from a baseline mean of 4.2 to a follow up-mean of 2.9 after undergoing eight weeks of therapy.
	Von Visger et al. (43)	14/0	Urban Zen Integrative Therapy	Intervention for anxiety only	Reductions in ratings of pain, anxiety, fatigue, and dyspnea symptom severity before and after the weekly UZIT sessions were reported (*p* < 0.0001). Symptom severity did not differ from week 1 through week 6 indicated that there was no cumulative dose/effect detected. Analysis indicated that about 50% of participants reported the absence of bothersome symptoms.
CTEPH	Vanini et al. (41)	70/0	Pulmonary endarterectomy (using moderate hypothermia and periodic circulatory arrest)	Intervention for depression and anxiety	Prior to surgery, mean baseline score of HADS-D was 6.11 while mean score for HADS-A was 7.70. Three-month score decreased to 4.48 and 5.95 for HADS-D and HADS-A respectively. Generalized linear p-value was calculated, and scores on both scales had significant changes post-surgery, with HADS-D (*p* = 0.002) and HADS-A (*p* < 0.001). Surgical factors and its association with depression and anxiety was also investigated. Anesthesia duration was concluded to significantly lower depression (HADS-D, *p* = 0.02) and anxiety (HADS-A, *p* = 0.08) scores. A longer total circulatory arrest (TCA) duration was also a significant relation to lower depression scores (HADS-D, *p* = 0.03)

*PAH, pulmonary arterial hypertension; CTEPH, chronic thromboembolic pulmonary hypertension; PMR, progressive muscle relaxation; QOL, quality of life; MCS, Mental Component Score; PCS, Physical Component Score; 6MWD, 6-min walk distance; HADS, Hospital Anxiety Depression Scale; PHQ, Patient Health Questionnaire; UZIT, urban Zen integrative therapy*.

## Discussion

The literature surrounding depression and anxiety in patients with PH remains limited. This is despite the European Society of Cardiology/European Respiratory Society guidelines, which recommend psychological support for all patients with PAH (2). This review summarizes the current evidence on the prevalence, risk factors, and interventions for depression and anxiety in PH patients, especially PAH and CTEPH. To our knowledge, this is the first meta-analysis to examine both depression and anxiety concurrently. The main findings of the study are (1) the relatively high prevalence of depression (28.0%) and anxiety (37.1%) across the countries, with significantly higher anxiety rates amongst PH patients in Asia compared to Europe and North America. (2) Significant risk factors of depression in PH patients were those with underlying PAH-CHD and CTEPH. The risk factors of anxiety in PH patients were underlying PAH-CHD, unfavorable pulmonary vascular resistance, and cardiac index. Importantly, traditional factors such as ethnicity, gender, and employment status were not significantly correlated to depression or anxiety. (3) Interventions for psychological adversity in patients with PAH and CTEPH have been systematically described and demonstrated useful outcomes in these patients.

There is a call towards an increased focus on mental health in patients with PH. This review highlights the alarming global prevalence of depression and anxiety in PH patients. Depression and anxiety not only adversely affects the quality of life (4), but have also been demonstrated to worsen prognosis in patients with other major comorbidities (44). Furthermore, two recently published studies corroborate the findings of this review. Takita et al. (45) performed a mixed-methods study, involving both quantitative and qualitative methods, and found PH patients to be more vulnerable to depression and psychological distress. Olsson et al. (46) similarly concluded psychological disorders, including major depression and panic disorder, to be prevalent in PAH patients and these disorders may contribute greatly to the patients' reduced quality of life.

This review found the pooled prevalence rates of depression and anxiety in PH patients to be 28.0 and 37.1% respectively across the globe, a stark contrast to WHO estimated global prevalence of depression and anxiety at 7.1 and 3.8% respectively (47). Comparatively, in a review of heart failure patients, the prevalence of depression and anxiety was 21.5 and 30% respectively (48). There may be possible pathophysiologic explanations to support the association between PH and the high burden of depression and anxiety. Perivascular inflammation plays a crucial role in the development of PH (49) and the elevated levels of pro-inflammatory cytokines such as interleukin-6 and tumor necrosis factor α have been linked to greater depression and anxiety (50). Antidepressants such as fluoxetine were found to reduce the levels of pro-inflammatory cytokines (51) and severity of depression and anxiety. Hence, antidepressants may have a protective effect on perivascular inflammation. PH development has similarly been linked to elevated circulating peripheral serotonin levels (52) which is strongly associated with the pathophysiology of depression. Serotonin can be synthesized in the pulmonary endothelium, released and pass into the pulmonary smooth muscle cells through serotonin transporter and/or stimulate serotonin receptors on the pulmonary smooth muscles to cause proliferation and/or contraction (52). Importantly, the use of selective serotonin reuptake inhibitors (SSRIs) as an antidepressant has been shown to block the serotonin transporter, leading to accumulation of extracellular serotonin, and enhancing the activation of serotonin receptors. As such, SSRIs have demonstrated an association with increased mortality and greater risk of clinical worsening in PAH patients (53). PH patients often face reduced QOL, difficult financial situations, and impairment of daily activities, all of which are significantly correlated with depression and anxiety (5).

Significant higher prevalence rates of anxiety were found in Asia compared to Europe and North America. This finding is the opposite to that of other epidemiologic studies, which revealed a lower prevalence of anxiety in Asian societies (54). Other studies have revealed that Asian populations tend to report somatic symptoms instead of psychological ones (55) and have a stigma associated with having a mental illness (56). Despite the higher prevalence of depression and anxiety amongst the Asian population in this study, Asians might continue to under-report psychological issues and under-utilize mental-health services (57). This suggests an underestimation of the true magnitude of the mental issue affecting Asians, reinforcing the strong impact that culture has on the expression and recognition of mental disorders (58). Notably, the small number of Asian studies available is a limitation. Greater attention toward the Asian population is required.

Another notable finding was that PAH-CHD was an independent predictor of both depression and anxiety. This is notwithstanding that PAH-CHD portends better survival outcomes compared to other subtypes of PAH (59). Early exposure to medical adversity, recurring periods of emotional distress, along with extensive anesthesia and surgical interventions were deemed sources of greater mental strain in CHD (60). Also, CTEPH was identified as a risk factor for developing depression. A study by Funabashi et al. stated how CTEPH patients might express a greater propensity for depressive temperaments even in the early stages of the disease, suggesting biological traits of CTEPH such as genetics to be a possible cause (35). Moreover, our study observed a trend toward increased prevalence of depression and anxiety in PAH associated with connective tissue diseases (PAH-CTD) though this trend was non-significant. One might expect a link between PAH-CTD and depression given the well-described increased prevalence of depression in the general CTD population. The underlying pathophysiology involves a dysfunctional adaptation of cytokine-induced sickness manifested from exacerbated activation of innate immune system and enhanced inflammation. This, in turn, contributes to clinical progression toward depression via dysregulation of the hypothalamic-pituitary-adrenal axis, adverse effects on neurotransmitter synthesis and reuptake (61). Future studies exploring the specific association between depression and/or anxiety and PAH-CTD are warranted to examine if this link between CTD and mood disorders is extended to PAH-CTD patients as well.

The findings of lower cardiac index and higher pulmonary vascular resistance being determinants of anxiety development might suggest that the above factors could affect cerebral hemodynamics and lead to depression (62). A study of CTEPH patients yielded analogous results, with the same two hemodynamic factors found to significantly correlate with the presence of psychiatric disorders (38). While hemodynamic severity appears to play a role in anxiety, there was no correlation with 6-min walk distance, functional class, or oxygen use, and we were not able to demonstrate that functional impairment was significantly associated with mood disorders (37). Therefore, clinicians need to be aware of this complex interplay between physical and psychological health, and not simply rely on their clinical judgment of the patients' risk of psychological adversity, mere symptomology, or functional impairment. Services for psychological support should be targeted at PH patients at greater risk of depression and anxiety, particularly those with CHD and CTEPH, as well as those with more severe stages of PH.

The risk of developing depression and anxiety in PH patients does not discriminate between age, gender, ethnicity, and socioeconomic status. Studies have shown that middle-income individuals are not at greater risk of developing mood disorders compared to high-income individuals (63). This trend was also observed in several studies examining depression and anxiety in CHD and chronic heart failure (64). Our study demonstrates that these traditional factors of psychological adversity amongst the general public might not have a similar effect on depression and anxiety in patients suffering from PH.

Published literature on interventions for depression and anxiety in patients with PAH remains scarce. Our systematic review aimed to demonstrate several of these interventions, all of which reduced depression and/or anxiety symptoms through the improvement of HRQOL (30, 41–43). Remarkably, slow-paced respiration therapy was found to reduce interleukin-6 levels (42), while MHCA during PEA significantly improved hemodynamics, motor speed, and even cerebral protection (41). These are compelling outcomes supporting findings of this meta-analysis, especially since hemodynamic factors such as cardiac index and pulmonary vascular resistance were found to significantly impact the odds of having anxiety in PH patients. Nonetheless, more prospective studies are needed to evaluate the efficacy of these interventions in PH patients with depression and anxiety. Furthermore, the implementation of the above interventions will require practical considerations, such as personnel expertise, equipment, and cost factors. However, with the focus of this review on the prevalence and risk factors of depression and anxiety, only four of the included studies discussed its management strategies without examining pharmacological treatments of antidepressants and anxiolytics. Nevertheless, it is paramount to consider the combination of pharmacotherapy and psychotherapy in this group of patients, as recommended by current guidelines with the use of cognitive-behavioral therapy (CBT) for moderate to severe cases, and psychotherapy as a valuable first step for mild depression and anxiety (65, 66). There remains a paucity of data on psychological interventions in PH patients. Further studies are warranted to examine the efficacy of pharmacotherapy and psychotherapy for depression and anxiety in this cohort.

At the 6^th^ World Symposium on PH, Simonneau et al. (1) proposed the lower criterion of mPAP >20 mm Hg for the diagnosis of PH, with suggestions that mPAP of 21–24 mm Hg correlates with increased morbidity and mortality (67–69). As most of the studies included in this review adopted the conventional criteria of mPAP ≥25 mm Hg, the overall prevalence of PH and the global burden of its associated depression and anxiety presented in our study, may be underestimated. Therefore, increased awareness amongst physicians regarding the vulnerability of PH patients to psychiatric conditions is key, and our findings emphasize the need for screening and management of these conditions.

### Strengths and Limitations

To the best of our knowledge, this meta-analysis is the first to examine the global prevalence, risk factors, and interventions for both depression and anxiety in PH patients. The heterogeneity of the included studies was a limitation that surfaced. To mitigate this, we carried out subgroup analyses based on the diagnostic tools used to measure depression and anxiety. In the measurement of heterogeneity, I^2^ values are known to tend toward 100% in studies involving large sample sizes (70). Additionally, the I^2^ can be an inappropriate measure of heterogeneity in single arm meta-analysis (71). Secondly, a large discrepancy exists between the number of studies included from each geographical region. Only four out of the twenty-four studies were conducted in Asia, with the rest localized in either Europe or North America. Hence, Asian PH patients might be under-represented in this meta-analysis. Furthermore, there was a paucity of data regarding the use of antidepressants and anxiolytics, which could potentially have a protective effect against depression and anxiety in PH. Hence, we were unable to perform meta-regression to evaluate for antidepressants and anxiolytics. Lastly, though this study reports on factors correlated with depression and anxiety in PH patients, further research is imperative to examine the causality and relationship between PH, psychological adversity and the role of comorbidity including substance abuse (72).

## Conclusion

In conclusion, the global prevalence of anxiety and depression in PH patients is alarmingly high, with an increased prevalence of anxiety in Asia compared to Europe or North America. Psychological support is warranted for patients with PH, particularly at-risk patients with underlying PAH-CHD, CTEPH, and severe PH disease.

## Data Availability Statement

The original contributions presented in the study are included in the article/supplementary material, further inquiries can be directed to the corresponding author/s.

## Author Contributions

NC and CN designed the study. AM, OL, YH, GK, GL, and CN acquired, analyzed, and interpreted the data. CH, RH, YL, IK, ET, JY, NC, and TL revised the manuscript. All authors contributed to the article and approved the submitted version.

## Conflict of Interest

The authors declare that the research was conducted in the absence of any commercial or financial relationships that could be construed as a potential conflict of interest.

## Publisher's Note

All claims expressed in this article are solely those of the authors and do not necessarily represent those of their affiliated organizations, or those of the publisher, the editors and the reviewers. Any product that may be evaluated in this article, or claim that may be made by its manufacturer, is not guaranteed or endorsed by the publisher.
